# De La Prostitution Dans La Ville De Paris, Etc.

**Published:** 1858-11

**Authors:** 


### NORTH AMERICAN

### MEDICO-CHIRURGICAL REVIEW.

NOVEMBER, 1858.

### Analytical and Critical Reviews.

ART. I.—*De la Prostitution dans la Ville de Paris,* etc. Par A. J. B. PARENT-DUCHATELET. Troisième Edition. Deux Tomes. *Completée par des Documents Nouveaux et des Notes.* Par A. TREBUCHET et POIRAT DUVAL. *Suivie d'un Precis Hygiènique, Statistique et Administratif, sur la Prostitution dans les Principales Villes de l'Europe.* Avec Cartes et Tableux. Paris: 1857, pp. 732 et 892. 8vo.

*Prostitution in the City of Paris,* etc. By A. J. B. PARENT-DUCHATELET. Third Edition. Vols. Two. *With the Addition of New Documents and of Notes.* By A. TREBUCHET and POIRAT DUVAL. *Followed by a Hygienic, Statistical and Administrative Notice of Prostitution in the Chief Cities of Europe.* With Charts and Tables.

*Prostitution, considered in its Moral, Social and Sanitary Aspects, in London and other Large Cities, with Proposals for the Mitigation and Prevention of its Attendant Evils.* By WILLIAM ACTON, M.R.C.S. Formerly Interne of the Venereal Hospital in Paris, late Surgeon to the Islington Dispensary, Fellow of the Royal Medical Society, etc. London: 1857, pp. 189. 8vo.

*The Greatest of our Social Evils: Prostitution as it now exists in London, Liverpool, Manchester, Glasgow, Edinburgh and Dublin; an Enquiry into the Cause and Means of Reformation. Based on Statistical Documents.* By a PHYSICIAN. London: 1857, pp. 344. 18mo.

*De la Prostitution et de ses Consequences dans les grandes Villes, dans la Ville de Lyon en particulier; de son Influence sur la Santé, le Bien-Etre, les Habitudes de Travail de la Population, des Moyens d'y Remedier.* Par A. POTTON, D.M., etc. Paris et Lyon: 1842, pp. 289.

*Prostitution and its Consequences in Great Cities, and particularly in the City of Lyons; its Influence on the Health, Well-being, Habits of Labor of the People; the Means of Remedying it.* By A. POTTON, M.D. Paris and Lyons: pp. 289.

SOME social evils press more heavily on one people than another; some seem to belong to particular periods in the history of a nation. Prostitution enjoys the bad eminence of being an evil and a crime, which has been continued from the earliest times down to the present day, in every country under the sun, in every stage of society, and among people of all religions—Pagan, Jewish, Christian, and Mohammedan. No angry sectarian can taunt the members of another sect with toleration of this evil, and claim for his own an exemption from its presence and effects. If there be an exception it is among the Quakers or Friends. Prostitution is denounced in strong terms by all religious bodies; but no one of them has come forward with any practicable or working plan for its prevention, or even systematically and earnestly set about lessening its frequency and mitigating its deleterious operation. Partial efforts have been made in this sense by benevolent and religious individuals; sometimes personally, more generally by the instrumentality of a Society; but with small, though, let us gratefully acknowledge, some beneficial results, as far as they went. The urgent necessity for reformatory efforts must be felt by those who have given any attention to the subject, whether it be studied in itself or in its indirect effects and collateral bearings. Prostitution, as degrading the human being below the brute creature, since it has not the excuse of animal appetite in those who follow it as a trade, is an enormous evil, which must be abhorent to the pure minded, as it ought to be revolting to the right sense of every reasonable being. But this vice stands not alone in its debasing character: by its spread it evokes or unites with other forms of evil, either as instigator or abettor. To prostitution are soon added lying, deceit, fraud, theft, drunkenness, and violence, which does not always stop short of murder. The physical ills keep pace with the moral ones; and we find a break down of health, incident to an irregular life and exposures to atmospherical vicissitudes, and which shows itself in various diseases. Foremost, by its frequency and destructive action on the organism, is syphilis, of which prostitution is the unceasing distributer in every direction through the community.

At the first view of the subject, it would seem as if retributive justice showed itself in the penalty for illicit intercourse between the sexes, by the disease in question; but alas! the innocent are made to suffer also, and the poison is brought from the brothel to the nuptial chamber, and contaminates the unsuspecting and virtuous wife, and through her the offspring. In a more insidious manner the mischief is effected by a man, who, after his constitution has been ruined by venereal excesses and disease, enters the married state, and becomes the father of children whose inheritance is decay and corruption of the springs of life.

The prevalence of the disease among the different classes of society may be inferred from the fact that nearly one-half of the surgical out-door patients of the London hospitals seek relief there for venereal affections, of which number about one-half are women and children. "One hospital alone, St. Bartholomew's, registered nearly 15,000 such cases on its books during one year."*

If the question be asked, how so deeply-rooted and wide-spread, and so virulent a moral pestilence as prostitution can be prevented, or even mitigated in its destructive operation, the reply can hardly be made in direct terms, or by a brief statement of means, unaccompanied by postulates and explanations demanded by the complexity of the problem offered for solution. Shall we reply in negatives, and say that the question cannot be satisfactorily met; but must be passed over in entire silence as a thing too gross and impure to be approached, or, if noticed at all, it can only be in terms of angry and general denunciation at the enormity of the vice, and the sinfulness of those who indulge in it? It may not occur to the mind of the stately matron, who has lived from childhood surrounded by all the aids to a virtuous life, derived from good example and associations, and religious instruction, and who would shun the very mention of the subject as carrying with it a taint, that the poor wretches whom she would treat as Pariahs, as outcasts, degenerated beyond redemption, and foul to loathing, may, some of them, have been seduced from the paths of innocence, and made what they are by the devilish arts and the lusts of a husband, a brother, or a son. Is she sure that the persons who are so dear to her may not habitually indulge in the mercenary caresses of these nameless and abhorred creatures? Were this estimable lady to be made aware, or had she even a suspicion of such transactions, antecedent or present, she would probably lend a pitying ear, if not at once to the voices of the frail ones, yet certainly to the narratives by others, of their wrongs and their misfortunes, and admit that their situation ought to be inquired into, and means devised for at least ameliorating it.

---

* Brit. and For. Chirurg. Rev., April, 1858.

We put the case thus strongly as an answer to the mawkish delicacy of some and the pharisaical righteousness of others,—the numbers of both of which classes are, we cannot but believe, diminishing in numbers as well as in influence. But a more cogent argument for benevolent and judicious investigation, with a view to abatement of the evil and reform of the evil-doers, than any based on selfish considerations, is found in the clearly expressed precepts of Christianity, and the benevolent example of its divine head and teacher. Wherever these influences are acknowledged, there must men, and women too, be found ready to aid in extricating from the trammels of vice a much-wronged, as well as wrong-doing portion of their fellow-beings. While performing these kind offices, they will at the same time find themselves engaged in co-relative efforts for preventing the extension, through the various ramifications of society, of a series of both moral and physical contagion which, without such checks, would corrupt to the very centre.

In France, and in Paris too, which is the epitome of all that is French, where every phase of society and every social problem is investigated and discussed with a breadth of illustration and a freedom of language which sometimes surprise the more cautious and reserved Anglo-Saxons,—English and American,—Parent-Duchatelet, the author of the first of the works which we propose to introduce to the notice of our readers, thought some explanation necessary for his selection of such a subject. The answer of this benevolent physician, who devoted so many years of his life to sanitary investigations, in disregard of his own health, and eventually at the sacrifice of his life, to his censors and advisers, was short and conclusive. It should be known, for the better understanding of his references to antecedent labors, that he had traversed every leading sewer of Paris, and spent hours at a time in them, in order to ascertain the better any faults of construction, and the means of rectifying them.

"If I could, without shocking any person's feelings," writes Duchatelet, "enter the public sewers of every kind, and come in contact with putrescent substances, pass a part of my time in the receptacles for all kinds of exuviæ and filth, and live, in a measure, in the midst of all that a collection of human beings exhibits of the most abject and disgusting nature, why should I be ashamed to approach a sewer of another kind, (a sewer more filthy, I admit, than all the rest,) in the hopes of doing some good, by examining it under all its aspects? By devoting myself to investigations on prostitutes, shall I then be necessarily corrupted by contact with those unfortunate beings?" The sentence which follows may help to undeceive those who are in the habit of associating with the French character, and especially with that of French women, ideas of levity, frivolity, love of exhibition and of personal adornment: "And if," continues Duchatelet,

"venerable ladies, who by birth and social standing belong to all that is most elevated among us, do not believe that they are lowering themselves by coming from time to time into the midst of prostitutes, for the purpose of enlightening and instructing them, when they are in prison, or in infirmaries, what have I, a mere individual, to fear in imitating them, and endeavoring to reach the same end, even though I should pursue a path somewhat different from theirs?"

Duchatelet persevered in his researches for a period of no less than eight years, and he has recorded the results in a work unique at the time of its appearance, and which not merely silenced all objections to the wisdom of his course, but has contributed more than anything that had been previously written to the better understanding of a great social evil, and to incite others to pursue the same path of inquiry. From the joint labors of the benevolent and indefatigable Frenchman, and those of medical and other writers elsewhere throughout Europe, there has resulted a full and methodized knowledge of the subject, which was absolutely necessary for the enlightening of those who might engage in the task of reformation, whether this were to be attempted by governments, by associations, or by individuals.

The subject of prostitution comes specially under the notice of the medical observer in its relations to public hygiene, and to pathology. Under another point of view, that of morals, he enjoys not only the common right, but he feels that he is enjoined, as if it were a part of his ethical code, to study it with care and to note the connection, in this as indeed in every other situation in life in which a human being is placed, of morals with hygiene. It is impossible to enter fully into a consideration of the one without continued attention to the other.

In the impossibility of doing common justice, in a single article, to a subject of such diversified aspects, and which deals so much in minuteness of description and multiplicity of details as the one now before us, we must content ourselves with pointing out some of the chief land-marks in this wide field, and trust to the chances, hereafter, of opportunity for further survey.

Prostitutes in Paris have been described by their historian with a detail and an impartiality with which a writer might be supposed to describe a new tribe of people coming from a far country and settled in the French capital. We read of the distant origin of many of them, the circumstances which induced them to leave their homes, their conduct in their new dwelling-place, their struggles to make a livelihood, the crimes added to the original trade, itself criminal, into which they were led,—their physiology, traits of disposition and character, social state, as measured by the society of which they are outcasts, and as displayed in the divisions among themselves, from the kept mistress, playing the part of a fine lady, down, through intermediate degrees of degradation, to the half-clad, frequently drunken and loathsome creatures, who hang around barracks for troops, and the vilest drinking-shops to which the refuse of the people resort, and who fall far below the nearly naked harlots of Central Africa, as described by Barth in his Travels. The historian follows the prostitutes into the prison and the infirmary, in both of which they are frequent inmates; he tells of their registration—certificates of their own infamy; their lodgings; restrictions imposed on them, and the sanitary precautions adopted for their benefit, and which serve, at the same time, to prevent their spreading syphilis among their customers,—for so one may term those individuals of the other sex who buy the temporary use of their persons.

The prostitutes of Paris may be fairly regarded as representatives of the type which is seen in all civilized communities; and hence, with some modifications, growing out of nationality and police restrictions, we may accept Parent-Duchatelet's* history of Prostitution in the City of Paris as an exposition of its leading features in the other cities of Europe and America.

The first question commonly asked, and which seldom obtains a satisfactory answer, when researches are made with a view to solve it, is, the *number of prostitutes* in any given place. On this point there is a general tendency to exaggerate even by those who, from their holding semi-official stations, might be supposed to be well informed respecting it. The editors of the great work of Duchatelet exhibit a tabular view of the number of registered prostitutes, or women of the town, in Paris, in 1854, which is set down at 4232, in a city containing a population of about a million and a half of inhabitants. It is impossible to form a correct estimate of the number of those whom our author calls clandestine prostitutes, and who, although engaged partially in some legitimate calling, make, nevertheless, a trade of their persons. These are variously estimated at 20,000, 40,000, 50,000, and even 60,000.

The question which follows that on the number of prostitutes in Duchatelet's work is,—*What countries supply Paris with this class of persons*, and in what proportion? Of the 12,707 women inscribed in Paris from 1816 to 1831, or during a period of fifteen years, thirty-one came from the three continents external to Europe, and 451 from countries in this last, other than France. We shall not enumerate these, but repeat

* The family name of the author is Parent; the addition of *Duchatelet* being derived from a country house called *le Chatelet*, to which his parents retired when he was two years of age. He died in Paris in 1836, aged forty-five years.

the observation made by the author, that the majority of the 451 females were supplied by the capitals of the different states. The regularity of the yearly arrival of these strangers in Paris merits notice. The same proportionate number, as we learn from the editors of the work, MM. Trebuchet and Poirat Duval, was recorded during the ten years between 1845 and 1854, and distributed very much in the same geographical ratio. When the inquiry is directed to the proportions contributed by the different provinces of France, the number collectively being 12,201, in a period of fifteen years, great inequality is observed in this respect. Omitting tabular details we reach the conclusion drawn by the author, and corroborated by his editors, that the nearer the several departments, in which France is divided, are to Paris, the greater is the number of prostitutes, or of those ready to become such by inclination or to gratify the vanity of dress, who resort to Paris.

The *social standing of the families*, some of the members of which contribute to the list of prostitutes in Paris, is the next point for examination by Duchatelet. The difficulties which stood in the way of his procuring the required data for forming a correct opinion in the matter, were overcome by his inducing the Minister to insist that all those who inscribed their names should bring an authenticated certificate of birth. As relates to the prostitutes born in Paris, it appears that a large majority of them were daughters of artisans; and that a similarly large proportion of those from the provinces were from the laboring class and those of stinted means. Parents thus situated are unable to attend to the education of their children, or to watch over them, and still less to provide for their wants when they have reached a certain age. Families in these circumstances supply house-servants and factory-girls.

From this point, by easy transition, the author passes to an inquiry into *the degree of instruction possessed by the fathers*, he does not say the parents, of prostitutes, as well as of the witnesses to the certificate of birth. Of 718 certificates of birth, of registered prostitutes, there were 545 signatures by the fathers, and 173 in which there was no such signature, owing to the inability of this number to write. Hence we see that one-third of the fathers of these girls were unable even to sign their names, and this in Paris, where primary instruction is accessible to everybody, and where it is deemed to be of the first necessity. In the departments, the ignorance of the fathers was still greater than in the capital. The inferences from these facts are seen at once, without commentary or argument: they point to the dangers, in a moral point of view, growing out of poverty and ignorance, as well as to the drawbacks to material comfort and prosperity from the same causes. Duchatelet says nothing about the religious instruction of prostitutes in early life, nor to what extent a want of its restraining influence contributed to hurry these unfortunates into their criminal career.

The *ignorance of the females themselves* corresponds with that of their parents. Of 4470 who were born and brought up in Paris, 2332 were unable to sign their names, 1780 signed, but in an imperfect manner, 140 wrote their names well, and some of them very well. No returns were made of 248 persons of the preceding total. We find, then, says Duchatelet, that in the capital of France, in which instruction has always been generally diffused, and is imparted gratuitously to those in indigent circumstances, and in which its importance is felt by the people, because it is necessary for obtaining a livelihood, there is but one girl with an indifferent degree of instruction for 2.3* who are entirely without it. Shall we say that these miserable beings were wanting in capacity, or that their parents were utterly indifferent to the subject? If this latter, do we not find a proof of the moral degradation and neglect in which parents allowed their children to remain; subjecting themselves to be reproached by these unfortunates for all the excesses into which they have been plunged? In the prostitutes supplied by the departments the proportion of those entirely ignorant was not so great as among the Parisians. On this point there is a want of agreement with what had just before been said of the greater ignorance of the fathers out of the capital.

A list is given of the different *occupations* of the women, numbering 3120, at the time of their entering the ranks of prostitution. A long one, embracing forty-one separate heads, is also furnished by the editors. It is largely made up of females who had been engaged in the various trades and occupations in which the needle is used: it includes of course seamstresses so-called, mantua-makers, milliners, embroiderers, shoebinders, makers of artificial flowers, etc. To these must be added laundresses, crimpers, etc. The majority, however, or eighty-seven in 1000 prostitutes, had no regular calling at the time of their inscription. House-servants furnished eighty-one in 1000. Next come depilators, so far as consists in the extracting of gray hairs. Of the entire number noticed by Duchatelet, there were three midwives; seven women who had kept shop and were doing a good business; one was a good landscape-painter; six were musicians and gave lessons on the piano; sixteen had been actresses or figurantes in the theatres of Paris and of the departments. Finally, and as a rare exception to the general rule, three had, respectively, a yearly income of 40, 100, and even 200 dollars. The author acknow-

* If the terms of the proposition were to determine the ratio of the ignorant to the whole number of prostitutes born in Paris, omitting the 248 of whom no record was made on this point, the answer would be one to 2.7, or nearly as three to seven.

ledges his inability to explain the motives which prompted these women to register their names in the general list of prostitutes.

The tables of the *ages* of the females who were inscribed as prostitutes in Paris show that some of them were only twelve years old, and others but ten and eleven years—a fact which, of itself, speaks most feelingly of the horrors of the vice of prostitution, when mere children are found to ply their trade in it. From sixteen to twenty-two years of age there is a gradual increase in numbers. At this last date the culminating point is reached, and the number declines every year, with some irregularities, from nearly seventy-seven (76.97) in 1000, at twenty-two years of age, to thirty in 1000, at sixty-five years of age. At the present time minors, or those under the age of fifteen years, are not received as subjects for inscription, and, if found leading a life of prostitution, they are at once arrested and sent home to their families.

The *first causes* of prostitution are, Duchatelet thinks, very varied; not being the same in the case of women of the cities and those of the country, nor among those from the provinces in general and those originally Parisian. He indicates the chief causes, as far as he could learn from the parties themselves, and as the result of other inquiries. He regards it as an undoubted fact, that all those women who give themselves up to public prostitution had previously led a disorderly life of longer or shorter duration. In the space of ten years, not more than three or four girls were seen at the Dispensary with the intention of having themselves inscribed, who had not lost their virginity. That there should be a single one in this situation, willing to pursue the vile trade without the excuse of previous betrayal, affords matter for melancholy reflection. Ought we not, however, to regard the disorderly life, which implies a loss of chastity, as a secondary rather than a primary cause of prostitution? It is the first step in the downward career, the causes of which, called secondary or individual by the author, we shall now specify. Foremost among these he places idleness, which brings with it the desire to procure pleasures without work. The idleness, recklessness, and want of energy of prostitutes, have become almost proverbial. Poverty, often carried to the most frightful extent, is likewise one of the most efficient causes of prostitution. How many young women, abandoned by their family, without relations and without friends, and unable to find refuge anywhere, are obliged to have recourse to prostitution in order to prevent them from dying of hunger? One of these unfortunates struggled to the very last extremity before she took such a step; and it was shown, when she had herself inscribed, that she had not tasted food for three days previously. Instances of this nature occur in every large city, and their number and character, as we shall show hereafter, if opportunity be allowed us, is frightfully great in London. Vanity and the desire to shine in rich attire are, together with idleness, the most fruitful causes of prostitution. The editors of the work before us do not hesitate to declare that these are traceable, generally speaking, to coquetry and an inordinate desire for luxurious indulgences.

We may say, in fine, that the love of dress and show exerts a much more seductive influence than any love that may be felt for the person who is regarded as the seducer, but who, in such cases, may accomplish his purposes without making vows of love and constancy. All that he has to do is to minister to the vanity and fondness for personal show of the silly girl. In fact, the intercourse between the parties becomes, without their avowal or even consciousness, an affair of trade, more prolonged and varied in its course than in the case of common prostitution, but it is essentially of the same character. The girl, hitherto innocent, sells herself for dress, but without specification of cost or terms, the very mention of which would shock her; the prostitute, more bold and frank, sells herself at a specified, and it might be called, a market price. The former of the two may obtain credit for a selection of her mate, a show of sentiment on the occasion, and even imaginary love, but her constant passion for personal adornment makes her accessible to any professed admirer who will gratify it. When a woman perpetually lays herself open to the seduction, whether it be of passion, of money, or of dress, the seducer, or rather the *particeps criminis*, is not long in presenting himself.

This is one of the causes of prostitution which ought to be pressed home to the mind and conscience of the young, and made to enter into the convictions of mothers, who so often encourage the love of dress in their daughters, and indulge in littleness of every kind to procure means for its gratification. Associated very generally with the love of dress is the love of amusements, and these, too often, of the most frivolous nature; and he, who, outside of the nearest ties of consanguinity, is allowed to minister to these loves, with the consent of the mother, or of the parents jointly, may be said to have, already, a mortgage on the person, if not a share in the affections of the daughter. The responsibility for the evil consequences which are too apt to ensue, must be divided in such cases. The man, on his side, does not often begin his intercourse with corrupt motives: he takes pleasure himself in the amusements to which he is the escort; he is gratified with being the companion of a young and pretty, or sprightly girl; he is sometimes the flattered as well as the flatterer; and, up to a certain point of time, which is determined by unexpected and unsought-for opportunity, following it may be the excitement of the giddy dance, or of theatrical exhibition, or still more of hot blood, wildly hurried in its round by intoxicating liquor, he had no thought, certainly no determinate thought, of the life-injury which, in a moment of forgetfulness and passion, he inflicts on his companion. If her subsequent fate be pitiable, his conduct is not always without palliating circumstances. We are telling now unpleasant truths; and we do so with a view of recommending habitual caution and forethought, and also of creating alarm in the minds of all parties at the very idea of a young and inexperienced girl being exposed to temptation in her taking a single step in a wrong or a doubtful direction.

The *fondness for personal display and of outshining their companions*, which so often leads to unchaste courses, and finally to prostitution, continues to display itself in an exaggerated style in those who have given themselves up to the trade. The editors of Duchatelet relate the case of a kept mistress who was allowed 600 dollars a month by her paramour; but this sum fell short of her wants, and, with a view of increasing her income, she went, from time to time, to a house of clandestine intercourse, or a house of assignation, where she was arrested. In reply to the observations made on her conduct, she said that she could not put down her carriage and do without her servants, and that she would prostitute herself to her coachman if he would engage to keep her horses.

The women of the provinces are exposed to a particular cause of prostitution which those of Paris escape. It is *desertion by their lovers*. A large number of young men,—officers of the army, students, and commercial travelers, seduce young girls in the provinces, contract a *liaison* with them, and by lying promises of marriage, or of forming an establishment for them, or through the necessity the girls are under of concealing their shame, carry them to Paris, where they are soon abandoned, and the poor creatures are left to work their own way. We may imagine their situation—entire strangers, left in a furnished lodging, sometimes in the street, and without money, in a city like Paris, and, to complete their misfortunes, not daring to show themselves in their natal spot, where their misconduct is known, nor to return to their families, which they have disgraced, and whose anger, and even hatred, they have incurred. Can one be surprised that a young woman, thus situated, should lend a willing ear to the suggestions and promises of those whom she meets? It is well known that on this class of girls do these detestable women, whose trade consists in corrupting and perverting the minds of youth, more particularly fix their eye, and track them everywhere; exhibiting in this infernal business a remarkable degree of address.

Some girls are compelled to leave their homes, owing to domestic troubles and the bad treatment which they have received from their inhuman parents. Sometimes they tell that they were driven to this course by the brutality of a stepfather or of a stepmother. Many, it would appear, are chased from under the parental roof, probably owing to their own misconduct, for there are parents thus barbarous, although we are bound to believe that the number is small. A long stay in a hospital, or in poorly furnished lodgings, to which servants out of place go, is often the immediate cause of the criminal course of many women—not so much from their being thrown into corrupt company as from being exposed to the machinations of those detestable procuresses already alluded to. These wretches employ agents who keep them apprised of everything that passes, and send them notices of all the girls who may suit their vile purposes. Many are not aware that there is a more wicked trade in living bodies, and in living souls too, than the African slave-trade itself. The misconduct of parents, and the bad example in every way which they set before their children, should be regarded as a determining cause of prostitution. The papers of such girls and the minutes of the interrogatories made to them, reveal, continually, domestic disorders; widowed fathers living in a state of concubinage; widowed mothers, and even married mothers, having lovers; their fathers and mothers separated, etc.

Thus privations, apathy, and the necessitous condition of many of the people of the lower classes foster, certainly do not guard against, and are powerless to prevent the corruption of their children. It may be said of a goodly number of prostitutes, what observation has shown to be true of malefactors—that they are for the most part of low origin. Confining our notices to young girls, what ideas of virtue can they have, when from their early years no attention is given to what they either hear or see, and when they find their parents (if indeed they be not natural children) separated from each other, and contracting adulterous relations? "Compelled, for the most part," says the author, "to take to the public streets at dawn, to sell fruit, vegetables or songs, or mixed up in the workshop with young persons of their own stamp, they soon acquire licentious habits, and form prematurely immoral connections (*liaisons;*) they have lost their innocence before nature has revealed herself. There *liaisons* cannot be durable, and the unfortunate creatures are already prostitutes in the midst of labor, and under the eyes of their parents. With such antecedents, is it to be wondered at that the sight of their comrades, already given to prostitution, that their idleness, always the companion of vice, and the reports which reach their ears, of the pleasures procured by debauch, because it allows them to gratify all their desires without recourse to labor; is it, I say, surprising that such a concourse of circumstances deprives a young girl of all power to resist seduction?"

Here is a picture largely suggestive to the minister of religion, the master manufacturer, the philanthropist, the political economist, and the statesman. It is one to be looked at not merely with an eye of sympathy, and expressions of pain and regret: it calls for action, at once systematic, practical and persistent. The author reverts to a fruitful cause of prostitution in Paris, and in all other large cities, viz.: "the want of employment and the destitution, which are inevitable results of inadequate wages. What," he asks, "do our mantua-makers, our seamstresses, and all those who are occupied in needle-work, earn?" If we compare the gains of even the most skillful with what can be done by those of moderate readiness in this line, we can see whether it is possible for the latter to procure the strict necessaries of life. Comparing especially the price of their labor with that of their dishonor, we shall cease to be surprised at seeing so great a number fall into a disorderly course, which would seem to be inevitable. The temptations from this cause, and the terrible suffering encountered by females, including daughters, and widowed mothers, and older sisters, before they yielded to them, are forcibly portrayed in the narratives of prostitution in London, of which, as representing, to a certain extent, a similar state of things in our own large cities, we shall say something more hereafter.

The lamentable picture of want, and of its effects on female character and conduct, receives some additional touches from the pen of the author in his notice of the encroachments by men on a large field of employment which it would be more just and honorable to leave to women. "Is it not shameful," he exclaims, "that we should see in Paris thousands of men, in the full vigor of their age, who lead, in coffee-houses, shops and stores, an easy and effeminate life, only suitable to women, and who are engaged in rubbing the dust off silver plate, or handling trinkets? The effects on them are ignorance, and, in a short time, enervation." The substitution of men for women in various kinds of business, although perhaps more common in Paris than in other places, is not by any means confined to that capital.

In looking at the great social evil of prostitution, Duchatelet very pertinently inquires, whether society has sufficiently occupied itself with the fate of those women who keep it up, and who, for themselves, claim its care, independently of the importance of the subject as a question for government action. Many ameliorations have yet to be made, but these cannot be attempted without an accurate knowledge of the evils and abuses which require correction, and the concerted efforts at reform by all the parties who have an interest, and to a certain extent, the ability in bringing it about. The fact would scarcely be credited, but it is, alas! too true, that the trade of prostitution has been taken up by some women as a means of enabling them to discharge the duties devolving on them as mothers or as daughters. "It is not," writes the author, "an uncommon thing to see married women, abandoned by or deprived of their husbands, and consequently left without any support, become prostitutes, with the sole design of saving a large family from starvation. It is still more common to find young women, who, not being able to provide for the wants of their aged and infirm parents, take up the trade of prostitution to eke out what is necessary."

The editors direct attention to a motive for prostitution which is more frequently operative than these just given. It is, to enable the mother to retain her child, for whose support her scant earnings are entirely inadequate, owing, in part, to the time taken up with her infantile charge. The following case, among others, will serve for illustration. A young girl, well brought up, lived with her mother, whom reverses of fortune had reduced to a state bordering on indigence. The mother, owing to excessive labor and prolonged vigils, became nearly blind. The daughter was seduced by the proprietor of the house in which she lived, and was afterwards abandoned by him. Unable to provide for the wants of her child and her mother, she went to the office and asked to be inscribed. As she was no longer a minor, her seducer could not be prosecuted for desertion; and representations made to him were without effect. The prefect of the police, M. Delessert, gave her some assistance out of his private purse, which retarded the catastrophe, but did not finally prevent it. This girl was not regarded with a favorable eye by others of the trade, with whom, be it said however, she would not associate. She contracted on different occasions many severe syphilitic affections; and on the death of her mother she disappeared, without its being known what became of her. In such a case as this we can form some idea of the dire necessity which drives a woman to prostitution, and forces her to continue it after having suffered again and again from a loathsome, painful, and disfiguring disease.

Some women, we are told by the author, give themselves up to prostitution in a spirit of utter licentiousness, which can only be explained by the supposition of their laboring under insanity.

Duchatelet introduces the question,—whether prostitution is owing to the *extreme degree of civilization* which is now reached? History and the observations of travelers give a decidedly negative answer, and the author justly remarks, that if our present social state be accessory to the loss of many women, this same state enables many to provide resources which they could not otherwise have had, and which allow of their preserving their honor, and of continuing in the path of virtue.

The causes of prostitution in 5183 cases are presented in a tabular form by Duchatelet: they indicate the proportions furnished by Paris and the departments, respectively, and specify, as regards the latter, the returns from the chief towns, the sub-prefectures, and the country. We shall not repeat these, but present the totals under the head of each of the separate causes. First in the list are poverty and extreme destitution, which number 1441; next, as far as relates to numbers, although it appears last in the table, is concubinage, by which kept women having lost their lovers, and not knowing what else to do, resorted to prostitution: they amount to 1425. The third, in the series of causes, is loss of parents, expulsion from the paternal home, complete abandonment, 1255; brought to Paris and abandoned by the persons already described in a preceding page, 404; servants seduced by their masters and then deserted, 289; those coming from the provinces to conceal themselves in Paris, and find means of living there, to support aged and infirm parents, 37; the eldest of the family, having neither father nor mother, with a view of providing for their brothers and sisters, sometimes their nephews and nieces, 29; widows destitute and abandoned, in order to bring up a numerous family, 23. Mention is made of some additional and revolting traits in this gloomy history, such as two sisters being inscribed together on the register of prostitutes; this occurred in 164 instances in the above grand total of 5183 individuals. In four cases there were three sisters, and in three cases, four sisters—in all 252 sisters. Besides these, there were sixteen instances of mother and daughter on the list; and twenty-two of cousins-german. The authorities have decreed that a mother and daughter cannot have their inscription together for the same house; so it is in the case of two sisters, if one of them be a minor. It should be stated that the entire number of the instances of close consanguinity between the 436 prostitutes, as above, must be spread over a period of seven or eight years.

The *manners and habits of prostitutes* are the subject of a highly interesting chapter in the work of Parent-Duchatelet. It is, indeed, as he justly remarks, one of the most important in the history of this class of human beings. How, he asks, in nearly the same terms which we have already made use of, when speaking of the causes of prostitution, can ameliorations be procured, reforms brought about—in a word, good done, without a knowledge of the tastes, manners, habits, and failings of the class whom we are studying? Curiosity will be felt to know what prostitutes think of themselves, and whether they are those hardened, reckless and shameless beings whom their deportment and speech, when seen abroad or spoken to casually, would seem to indicate. Studied at other times, as when they are in prison and suffering, and particularly when, by proper measures, their confidence is obtained, we can discover what passes in their minds, and how oppressively they feel the weight of their infamy. We are authorized in saying, that they know their course to be an evil one, and that they are justly despised; and hence they never feel at ease except when they are in each other's company and with other depraved persons. The sight of mothers of families and of virtuous women is hateful to them, and they take pleasure in insulting such persons, in revenge, as it were, for the contempt with which they are themselves visited. Although, in the exercise of their trade, they affect a bold and forward air, many of them, under other circumstances, take great pains to conceal their true character, and dress themselves with remarkable simplicity. In their visits to the Dispensary they do their best to escape notice, and they may be said to glide in and out. Some of them coming from the provinces will choose very retired quarters for their residence, so as not to be seen by those from their own part of the country. In general, there is nothing that they dread so much as meeting those whom they had known before their lapse into sin. The author has seen patients in the hospital who became ill from strong emotion produced in this way. Much, in such cases, must depend on temperament and the degree of intelligence of the parties. We have ourselves known an instance of utter insensibility in a prostitute on her adverting to the very first step in her undoing. A message was received to visit a person who was represented to be very ill at a designated house in this city. The physician thus sent for gave prompt attendance, and on his entering the sick room he found two young women, one of whom was in bed. The latter, who was the patient, began giggling the moment the door was opened, and continued to do so after the physician had taken his seat. On his expressing a desire to know the cause of her merriment, she replied, in a subdued voice, "You put me so much in mind of the person who seduced me." Whether the poor girl told the truth, or had been laughing with her companion at some joke before the doctor's arrival, could not be ascertained; but, in either of the two suppositions, we have proof of the indifference with which she could advert to what might be called the great event of her life. She was at the time suffering from syphilis, to be cured of which she had sent for a professional adviser.

But "prostitutes," to resume the remarks of Duchatelet, "not only are aware of, but have a profound conviction of their abject state, and are objects of horror to themselves; the contempt with which they regard themselves exceeds that with which they are regarded by the virtuous portion of society; they mourn over their fall, and form projects, and even make efforts, fruitless though they be, to change their condition; and what makes them despair is their knowledge that in the eyes of all the world they pass for the very scum and refuse of society." "One of them, in conversation with a physician of the Dispensary, told him, in an effusion of confidence, that she would not attach herself to one man in particular, because, whenever she embraced him she would believe that she was sullying him by her contact. One day, finding myself in a ward of the hospital without my being seen, I heard a girl exclaim, while she was admiring the beauty of the sky, 'How good is God to send us such fine weather. He treats us better than we deserve!' And the entire ward repeated, at the same moment, 'That is quite true.'"

The very thought of their fallen nature has driven many prostitutes into mental derangement. One of these was pointed out to the author by Pariset at the hospice of the Sulpetrière, (the asylum for insane women.) She said nothing in public; but when she believed herself to be alone, she repeated, without ceasing: "How unhappy I am for having forsaken virtue! How can I bear the general contempt! How live under this humiliation!" This feeling of their abjection and of the contempt which they inspire might be said to excite still more their pride and self-love, faults very noticeable in their character, and the wounding of which insures their perpetual enmity. But, when spoken to with mildness, and in a manner evincing interest in them, and if hopes are held out that they may once more enter into society and regain their place in public good-will, their hearts palpitate with joy. Some incidents are related to show this peculiarity in the character of prostitutes. When the sick of their number were first sent to the *Hospital la Pitié*, there was no chapel on their side; and when it was erected they were excited to the greatest pitch of gladness. Not that they felt any religious sentiment on the occasion, but it showed that, to use the language of one of them, they were no longer regarded as dogs, and that the same was done for them as for other people. A physician completely won their confidence, and could make them do whatever he wished, and thus secured the most perfect order in the wards, simply by an act of minor politeness in slightly raising his hat on his first entrance. Another physician, who affected the greatest disdain for them, was not near so successful in securing discipline in his wards. To this feeling of pride is due the contempt which the different classes of prostitutes have for those below them in the scale, and the hatred with which the inferior classes regard those who eclipse them in grace and beauty. Some not over-cynical person may ask, whether this last trait is peculiar to the degraded of the sex, and whether, to use a geological phrase, it does not crop out on the surface among womankind generally?

The *religious sentiment* is entirely wanting in a large number of prostitutes, to such an extent, indeed, that some of them are ignorant of a Supreme Being. The most numerous examples of this nature are found among those who had been thrust into the career of vice by their parents, or left to themselves from early infancy, or who did not even know where they came from. In the exercise of their trade, and in their conversations with men whom they meet in their walks, they are not sparing of jokes and ridicule on religious matters, but when alone or in prison, it is not always so. In the streets, when free, they do not fail to cross themselves on the occasion of their meeting a funeral procession. In the infirmaries of the prison, in which they are often collected in large numbers, they do not refuse the services of religion in their last moments; and their companions all assent to the propriety of such a proceeding, and declare that, under the like circumstances, they would themselves do the same thing. They will not, either in the hospital or the prison, attend the chapel on compulsion, but if the doors of the chapel be open, and hymns and canticles are sung in a language which they can understand, they will crowd in and comport themselves in the most irreproachable, one might almost say edifying, manner. A girl in the lowest of the class, who lost her child after a long sickness, used to offer up prayers regularly during the whole time. On the occasion of the death of a prostitute at her domicile, all her comrades subscribed to procure for her remains a grand funeral service, and to pay for a great number of masses. Another girl, of an inferior order in the class, having died, her comrades, clothed in white, accompanied the body to the church, and placed around it "a *prodigious number of wax candles.*" We repeat those incidents as part of the history of prostitution in a Catholic country, and without feeling ourselves called on, in this place, to criticise, in a Protestant spirit, the validity of certain ceremonies. In anticipation of the evidences of benevolent feelings entertained by these unfortunates, and which are adduced by Duchatelet, we shall introduce here an example taken from the other side of the channel, in London, because of its direct connection with what has been said above of the religious sentiments of the French prostitutes. The writer,* after quoting Duchatelet's account, relates the following:—

> "A very touching instance of these amicable feelings was related to us a short time ago. A poor girl, who, after a few years spent in infamy and wretchedness, was rapidly sinking into a decline, had still no means of livelihood but in the continued practice of her calling. But with a mixture of kindness and of conscience which may well surprise us under such circumstances, her companions in degradation resolved among themselves that, as they said, 'at least she should not be compelled to die in sin,' and contributed out of their own poor and sad earnings a sufficient sum to enable her to pass her few remaining weeks in comfort and repentance. This is not a trait of the wholly lost."

That, in their ignorance, prostitutes should have wrong notions of the purposes of prayer will excite no surprise. Some of them have had masses said to save their lovers from the conscription, or to bring back those who had abandoned them. The belief that Friday is an unlucky day is general among them; and hence there are fewer inscriptions and fewer visits to the Dispensary on those days. Every girl who is not satisfied with the state of her health, and who is afraid of being sent to the

* Westminster Review, July, 1850; article on Prostitution.

hospital if she be thought sick, will never arrange it so as to have herself visited on Friday. We are indebted to the editors of Duchatelet for the following additional particulars. The respect of prostitutes for the clergy is very great, and when, owing either to ignorance or the kind of house to which he is taken, or to the danger of removing the patient, the priest visits a "*maison de tolerance*," or permitted brothel, the girls in it manifest extreme gratitude, and even veneration. Although, for the sake of humoring the men with whom they consort, they may sometimes join in ridiculing or aspersing the priests, yet they rarely speak ill of them, or seek to affront them in the public street. If, by chance, a priest should, in ignorance, take lodging in a house inhabited by a prostitute, or the latter come to live in a house in which a priest resides, she will immediately cease to carry on illicit intercourse at the house, and will remove as soon as possible to another quarter without waiting for any formal intervention of the authorities. The remembrance of early religious instruction which some of the prostitutes may have recived is never effaced, and speaking to them of this instruction and of their first communion never fails to make them weep.

Do prostitutes, notwithstanding their vices and their habits in general, *retain any remnants of modesty?* is a question to the reply of which the author devotes a section of a chapter. He gives it in the affirmative, and sustains his belief by several incidents which are to the point. Of this nature are the reserve and guarded conduct which they display before respectable women, who are at the same time mothers. Very many of them blush when they are obliged to expose their persons before a number of people, and, by an instinctive movement, they close their eyes on such an occasion. Duchatelet tells of his having followed the clinical course of Michael Cullerier at the *Hospice for Venereal Diseases*, and of his not forgetting the profound impression produced on the prostitutes by an examination and demonstration of their diseases before a large assemblage of students. In every instance, without excepting even the most hardened among them, they blushed crimson; they hid their faces, and regarded as a true punishment the trials to which they were thus subjected. A fact like this cannot but serve as a reply, we ought rather to say a rebuke, to those who quote French usages as an example, and assert that there is no harm in exposing the persons of female patients to a class of students in what are called Obstetric Clinics.

Parent-Duchatelet, writing in 1836, points out the great improvement during the preceding twenty years, and still more in the ten years which had just closed, in the deportment and manners of the prostitutes of Paris, even to the extent of bordering on modesty. Much of this change is due, he thinks, to the pains taken by the administration which exercises continual watchfulness and great perseverance in following out every plan of repression and reform. Prostitutes remain, in general, but a short time in their trade; going through it as a transition state, traditions are lost and easily forgotten by them.

The practice of *tattooing* is common with a certain number of prostitutes, and more especially with those who consort much with soldiers and sailors, whom they hope to please by imitating them in this particular. The skin is seldom marked or painted in this fashion on parts habitually exposed, but generally at the upper part of the arm over the deltoid muscle, under the mammæ, or on the chest. Almost always the tattooing consists in inscriptions, or in the proper names of individuals, followed by the words *pour la vie*, or the abbreviation P. L. V. Often these inscriptions are between two little flowers, or between two hearts, united by an arrow which runs through them. It may be worth while for those who study the odd exhibitions of human nature, to know that these names vary with the age of the woman. If she is young they are most commonly those of men; if she has reached a somewhat mature age they are generally those of women; and in the latter case they are invariably marked on the space between the pubes and the navel, which is never done in the case of the names of men. As the vow of constancy for life, implied in the inscription previously given, is soon exhausted, and as each new lover is complimented with a similar formula of devoted attachment, these women find it necessary to efface the old tattooing before the new is practised. This they contrive to do with great readiness by a simple chemical process which need not be given here. In one woman, not more than twenty-five years old, who was in the prison of the Madelonettes, no less than fifteen cicatrices, left by successive erasures and loss of cuticle and corion, could be counted. In one instance, the attempt to efface a name on the bend of the left arm cost the poor creature her life by an inflammation to which it gave rise. What merits notice is the absence of all impropriety or indecency of word or sentences in these inscriptions. The same may be said of the allegorical and other figures traced on the skin by a similar process. If exceptions were found to this reserve it was in girls of the very lowest order, who did it to please the gross tastes of the men with whom they associated. Even among this description of women these offensive maculations are now rarely met with.

A question of considerable moment presents itself as to the *manner in which prostitutes pass their time* when not in search of victims or purchasers, or otherwise engaged in their trade? As the author justly observes, there will be in this matter great differences, depending on class, and individual character and temperament. It may be said, however, that nine-tenths of them pass their time in entire idleness. Those of a higher order rise late, go to the bath, drink, eat, and romp, or throw themselves negligently on their bed or sofa: in summer they take promenades. Others spend their time in low wine-shops or taverns, drink and eat like the first-mentioned set, and hold conversation with vicious persons who frequent vicious places. Some of the better order work in embroidery or articles for the toilet, flowers, etc.; a few read, and a still smaller number have recourse to music. Some, in the second category, work at a trade or in factories, or sell articles in the street. This last occupation is generally preferred to any other. We should little anticipate the kind of reading to which they who pass any time in this way are most partial. It is a narrative of tragic scenes capable of exciting strong emotions. But, the most surprising feature is, that they have never been found with those licentious and obscene works which too many young persons are eager to peruse, as if they were under a necessity of taking lessons to prepare themselves for the trade of debauchery. All prostitutes, to whatever order they may belong, are fond of dancing, and in Paris they have regular balls near the barriers or in the adjoining villages. Paris, as the author remarks, is the place for contrasts. If the approach of evening be the signal for the greatest number of prostitutes to ply their trade, there are others who pursue it all day; and some even restrict themselves to a certain period of the day, after which they are no longer "at home;" and they pass the evenings in the company of their chosen lovers, or they go to the theatre, or to balls or concerts.

The *fictitious names* taken by prostitutes is the subject of a section in the work now under notice. Changes of name may be explained in many cases by some remains of shame, which would make them loth to be recognized by former neighbors and acquaintances; and also to avoid exposing their family. Another motive would be to conceal themselves from judicial pursuits for infractions of law, or from the surveillance of the police. For several years past, they are compelled, before being inscribed, to produce a certificate of birth, by which their real names are known. In the period between the years 1828, when this condition was made peremptory, and 1831, rectifications in the baptismal and surnames of these women were made to the number of 2271, or nearly one-half of all who were inscribed. The author gives a list of the *noms de guerres* which they assumed, or which had been given to them by their companions. The upper class show a great many names of a poetical or sentimental character, such as Armide, Calliope, Olympe, Flore, Malvine, Virginie, Delphine, etc.

The *filthy habits* of prostitutes, as evinced in their utter neglect of personal cleanliness, is one of their distinctive traits, a fact somewhat at variance with what might be supposed to be required by policy, as a means of increasing their attractions. The women of this class find a positive pleasure in dirt and filth; their only thought being of the external garments worn for show. When not, as it were, in a regular campaign, and no longer under public notice, they care not what tatters and rags they may have on; they exhibit no desire to receive clean linen; and it is only at the very last pinch that they will wash that which they have. This extreme of uncleanliness prevails most among the regular tenants of a recognized brothel, (*maison de tolerance,*) who, notwithstanding, rival in their toilet, as far as relates to show and elegance, whatever is most admired in general society. The intervention of the authorities has contributed greatly to diminish this neglect, both of decency and hygiene, and now, in many instances, it is replaced by ablutions which might be thought excessive, but which are particularly called for in prostitutes, both on account of their sex and their trade. After hearing of the filthy habits of these women we are prepared to learn that they are very subject to the itch, and that less than half a century ago their bodies were infested with vermin. Parasites on the head are still common in the young girls who are deemed the most stylish of their class.

Duchatelet denies that the prostitutes of Paris have a particular slang or jargon (*argot*) by which, like thieves and pickpockets, they can communicate with each other. They adopt certain expressions, but in small number, which are peculiar to them, and which they use when they are together. The case is different with those women who prostitute themselves to disguise their true calling, that of colluding with and aiding regular thieves, whose slang they adopt.

The peculiar failings of prostitutes, putting aside, of course, the vices inherent in their vile trade, are described by the author to be *gluttony, and fondness for wine and spirituous liquors.* Their gormandizing and voracity are extraordinary; some of them are continually eating, and consume enough for three or four women of their own age. They contract this habit from the disorderly characters with whom they make up parties to low eating houses, or even to those of a better stamp, according to the order to which they belong. The love of these women for spirituous liquors is general, although of course in different degrees; they acquire it very early, and with some of them it ends in the lowest degree of brutality.

All the investigations of Duchatelet prove that these women begin to drink for the purpose of deadening their sensibilities; gradually they get accustomed to it, and before long the habit becomes so fixed as to present a barrier to their return to virtue. In many cases it alone nullifies all the reformatory efforts of benevolent ladies.

This description of the progress of the habit of drinking, which leads to confirmed drunkenness among the prostitutes of Paris, is applicable to women of the same class everywhere, and, unfortunately, we must add, to women of a different class and merit in society, who, in order to obtund their griefs, caused by a drunken husband, or by other domestic crosses, have recourse, at first occasionally, to the bottle. A case illustrative of the additional dangers to which a female is exposed by the use of intoxicating drinks in her downward course to prostitution, and before she has regularly entered on the trade, came under our own observation many years ago. A beautiful girl, who, as she said, had been seduced by the son of an elderly lady in a neighboring State, with whom she was living as a dependent companion, came to this city. Here she soon "took up" with a young man, at whose request, as his professional adviser, we called to give her medical aid. She became pregnant after the second *liaison*, but whether she had been so after the first we did not learn. During this time she had gonorrhœa, from which she soon recovered. The child was, for awhile, in the hands of a wet nurse, and was afterwards put under the care of persons professing religion, but who were shamefully negligent of their trust. Happily, we ought perhaps to say, the child died in its second year. The mother, after the birth of her child, became addicted to the use of alcoholic drinks and narcotics. In one of the fits of delirium and stupefaction produced in this way, she wandered at night out to the commons, where she was found by persons, strangers to her, but who succeeded in discovering some of her acquaintances, lost characters like herself. This exposure and its concomitants cost her an attack of cholera, from which she recovered. She was now in a measure separated from the father of her child, and there was reason to believe that her conduct was anything but chaste. In the autumn of the year, she contracted bilious remittent fever, most probably from night strolls beyond city limits, and we were again called to visit her; this time at the house in which her child had died. Well do we remember the sore distress of this poor girl, not only from the raging of fever, but the stings of remorse. On one occasion she exclaimed: "If I die as I am now, I shall certainly be damned." She did die.

Prostitutes, as may have been already learned, are not "evil all." They have their good qualities, as Duchatelet shows, and as most medical men generally have had an opportunity of knowing. One of these is the readiness with which they aid one another in their troubles and misfortunes. They will stint themselves to help others of their class, who may have even previously deceived them, and from whom they expect no gratitude. Nor is this benevolence confined to one another; it is manifested often in their assisting the needy in their neighborhood. When they become mothers the better traits of their nature are brought out in strong relief. They find in the attentions required for their children an enjoyment which compensates them for the distresses inherent in their trade; and they look upon their new dignity, in being mothers, as elevating them in their own eyes from the abject state into which they have fallen. Notwithstanding the unquestionable love of these women for their children, the administration has wisely enacted, both for the benefit of mother and offspring, that when the latter have attained the fourth year of their age they shall no longer remain in the domicil, either of the mistress of the house or of the mother who lodges in it. A prostitute, when she is pregnant, becomes immediately an object of tender sympathy among all her companions; but the evidences of this feeling are accumulated at the birth of the child. It is then who shall wash the child's linen; who shall nurse the mother; who shall hasten to despoil themselves for her benefit. If the child survives there is no want of a cradle,—the struggle as to who shall have the little being is carried so far that the mother seems to have no direction in the matter. It is an undoubted fact, we are assured by Duchatelet, that prostitutes who have children are much more disposed to keep and nourish them, than girls who are mothers for the first time after their fall from virtue, and even than many married women.

The fate of the children of prostitutes is adverted to by Duchatelet, but he is unable to furnish precise figures on this point. Great numbers of them die. Some of those who survive are well cared for by their mothers, who either place them out of the reach of their own moral contagion, or are very guarded in their deportment before them. Instances of quite an opposite nature must of course be expected to occur.

The relations of prostitutes to the *lovers and bullies* whom they select for their companions, are described in the present work. The author lays it down, as an invariable rule, that, if libertinage and the fervor of the passions are the first cause of prostitution, so soon as these unfortunates enter regularly on their new career, they are cold and indifferent to all those who approach them, even to a feeling of disgust which may be hidden under the caresses they lavish for the love of gain, or often to satisfy the cravings of hunger. We shall be the less surprised, therefore, at finding some of these women attach themselves in a special manner to one man, and thus trying to fill up the void in their hearts left by the wretched life which they lead, and the remorse which afflicts them. The social position of the lover must necessarily vary as greatly as the division of the class to which the girl herself belongs. Letters found in the prison and hospital, and appeals made to the proper authorities in their favor, exhibited the melancholy fact that among the lovers of these "women of the town," were persons who not only moved in good society, but who by their name and position could not fail to excite surprise when they were found to be compromised in such affairs. Figuring on these occasions were seen the general, the man of letters, the peer, and the financier, and successively all the other classes, down to the lowest in the social scale. Sometimes men of note, whose names are familiar to the public ear, have been seen at the bureau of the prefecture, asking for the discharge of these women, and defending and pleading their cause against the administration. This scandalous exhibition was chiefly noticeable in 1817, when it was intended to subject women of pleasure, (*femmes galantes*,) of a certain order, to a sanitary regime. At the time of Duchatelet's writing these cases were of rare occurrence. For the most part, the higher order of prostitutes choose their lovers from students of law and medicine, and from young lawyers,—being induced to do so by the greater pleasure they derive from persons of cultivated minds; but the number of women of this order is small compared with the mass of those of the town. The middle order of prostitutes enlist their lovers from the ranks of merchants' clerks and of tailors, both of whom constitute a numerous class in Paris: to these we may add apprentice hairdressers, itinerant musicians and others of this class in the small drinking-houses of the suburbs; also jewelers and goldsmiths. Below these again, are the women who form a connection with workmen of every kind, bad fellows who are found in every crowded community, and more particularly in large capitals, the peace of which they are ever ready to disturb, whether this be done by ordinary breach of law or by participation in political revolutions. One of the phases in the life of prostitutes which merits most study is the extreme attachment evinced by them for their lovers, from whom they often obtain no money, but whom they frequently supply in this way as well as feed and clothe, out of the gains of their trade. The return which they are apt to receive for all these practical demonstrations of regard is abuse and bad treatment, extending to bodily injury and sometimes to severe wounds. Extraordinary examples of this nature are related by the author. The letters of the prostitutes when in prison exhibit the intensity of their passion for their lovers: these missives breathe an exaltation of feeling and imagination, but never contain anything smutty or vile. Sometimes they take vengeance by inflicting bodily chastisement on those who have supplanted them, and a few even assault their lovers who have abandoned them.

The lovers, so called, act the part of bullies for their girls, to whom, if on forbidden ground, they give notice of the approach of inspectors, or they try to extricate them from the hands of these latter, by making a disturbance and collecting a crowd so as to get the girl away, or failing to do this, they come to blows with these officials. In addition to the direct robberies which the bullies commit, they plant themselves in low taverns and drinking-shops together with prostitutes, foment all kinds of disorder, and trick or frighten novices into paying for the whole company. In fine, they convert these houses into a species of fort, in which they set the authorities at defiance, and sometimes are only expelled by the muster of two brigades of inspectors. These wretches, constituting themselves avengers as well as defenders of their favorite prostitutes, are always ready to play some ugly trick or another, and even inflict severe bodily injury on informers and others who are instrumental in checking the disorders or disturbances made by these women, or in getting them sent to prison for robbery. These bullies are a real leprosy in the community, which the authorities cannot reach, unless they were allowed the same supervision over them which is given in the case of prostitutes. One of the most sad and sickening comcomitants of prostitution is this connection between the women who follow it and their lovers so called. Strange and revolting love, when the woman is ready to prostitute herself to any passer-by, and yet all the time professes and really feels a strong attachment to one man; and this man consents to pass as the chosen favorite, and calls himself the lover of a woman, who supports him by the sale of her person promiscuously to others!

The unnatural passion which some prostitutes have for individuals of their own sex is a subject which the author could not dispense with noticing. He speaks of it with a reserve which we shall imitate, and pass on to other matters, after a mere mention of the designating title of the guilty women—*tribades*—and the remark, that the vice is said to be contracted in the ordinary prisons. This would be one among the many arguments in favor of the system of separate or cell confinement. Holding in mind the necessity of separation, an ordinance was issued in 1824, and is still in force, forbidding the keepers of brothels to allow two of their girls to sleep in the same bed.

A section is given to a notice of the *different classes* of prostitutes, the details of which would constitute desirable and even necessary information for those who might be authorized to take measures for subjecting these women to special police and sanitary regulations; but as the question of repression by such means is not a practical one under the existing laws of the United States and England, we shall touch lightly on it for the present. It will come up hereafter if an opportunity be given to inquire into the whole subject of amelioration and possible prevention. Of the different orders of bad women made by prostitution, the procuress is the most dangerous, because often not suspected, and the most mischievous, because she is continually on the alert to decoy inexperienced and innocent girls into the dens of infamy, under the plea of finding for them a safe and pleasant asylum until they can procure employment. Often she is the direct medium between a lecher and a girl, who, under various pretexts, is deluded into his embraces. Sometimes she is a broker who settles the conditions on which the man and woman meet. The most common disguise, in the way of regular business, which she assumes in Paris, is that of a dealer in articles for the toilet. In this capacity she finds access to every house. Purchasing only second-hand things, she is visited by women of all classes; some to sell what has been given to them, others to dispose of what they have stolen. Availing herself of the opportunities thus furnished, she is ever ready, with wicked counsel, to lead them into disgraceful situations. Some procuresses have their specialties, directing their attention only to one class, such as opera dancers and actresses; others carry on their business on a larger scale, and keep up a correspondence with the provinces and foreign countries, and send their victims to London and Brussels. Although they profess to have themselves abandoned prostitution, little reliance can be placed on their professions, and, at any rate, in their new calling they do infinitely more mischief than they could by their being merely prostitutes.

Walkers (*marcheuses*) constitute another division of those who, having given up the trade of prostitution themselves, still do service in the vile cause,—become messengers in brothels, and companions to the young girls of the house when the latter go to the bath, or to the prefecture of police. In the evening they stand at the door of the house (of ill-fame) and serve in place of a sign-board, to indicate its character to the passers-by. As apparently grave matrons escorting young girls, they enable these latter to evade the prohibition against their walking in particular parts of the Boulevards, or other frequented promenades.

The author prefaces his account of the different divisions of recognized prostitutes by some notice of a separate category of women composed of what are termed first, *Femmes galantes*, and secondly, of *Femmes à Parties*. The first are kept mistresses, whose expenses run beyond their allowances, and who, to increase their income, think themselves obliged to have sexual dealings with a certain number of the public. They profess great modesty and reserve when abroad, but contrive by a certain look or manner to indicate their true character to the initiated. They allow themselves to be escorted and followed; but, commonly, they receive the desired class of visitors at the houses of their friends, or at some private establishment. The second class do not rely on their personal charms alone, but enhance these by graces of manner and cultivation of mind. In order to obtain admission to their houses, it is necessary to be introduced by a regular visitor or *habitué*. They give dinners and suppers, and are invited to private saloons, to which company is attracted by the allurements of gambling and freedom from all moral restraints. Both these orders of women are, as Duchatelet very properly observes, prostitutes; but as conforming themselves to all the external observances of propriety, they escape the supervision of the administration, and may even sue for damages for slander if they are called prostitutes. They belong, notwithstanding, to the same scale as the regularly inscribed, including the miserable creatures who have no regular abiding place, and who are called girls of the barriers or soldiers' girls. These latter hang about the barracks and low drinking-shops of the suburbs, and, both physically and morally, may be regarded as the very dregs of the lowest and most turbid part of society.

A chapter is given to the *condition, physiologically considered, of prostitutes.* The plumpness or obesity of this class, and their fine, healthy appearance, strike all those who look at them in groups. There are, however, numerous exceptions; and one meets every day, women who not only exhibit nothing remarkable in these respects, but who are thin and even emaciated. It has been observed by persons who live among these women, that they rarely show embonpoint before they reach the twenty-fifth or thirtieth year of their age, or when only debutantes in the trade. There were not wanting persons, and some of them medical men, who believed that this fleshiness was due to the large use of mercury for the cure of syphilis, with which prostitutes are so frequently attacked. The better founded, and no doubt the true explanation, is the abundant alimentation and the indolent life of these women, and their frequent use of the warm bath, to which must be added their indifference to the future,—a frame of mind which one could not anticipate in this class of human beings. There are, of course, among them, as in the community at large, some ungrateful bodies which will remain lean in spite of all the means best adapted to give embonpoint. The prostitutes who go to the hospital, and those who are sent to prison, recruit their health, and come out less lean than when they entered. The remark has been made, that all classes of prisoners, even those condemned to death, gain flesh—a fact explicable, it is thought, by the regular life which they lead, added to the detention itself. If embonpoint be common among prostitutes, it is still more so with *dames de maisons*, or ladies so called of tolerated brothels, whose adipose development is truly remarkable.

A *peculiar alteration of the voice* has been noticed in prostitutes. There are some of these women remarkable for the beauty and freshness of appearance, their carefully arranged toilet, and the elegance of their manners, and who, by their carriage, one would take to be persons of refinement, possessed of everything that is pleasing and seductive. But what a disenchantment when they speak! In place of the quality of voice which adds to the charms of woman, there issues from their mouths nothing but harsh and discordant sounds, which crack the tympanum, and which a drayman could barely imitate! This harshness of voice, like embonpoint, does not commonly show itself before the twenty-fifth year of their age: it is most frequently met with among the lowest order of prostitutes who are loungers about petty taverns, and who, when intoxicated, are in the practice of uttering loud cries and vociferations, and also among those women who have fallen from the first to an inferior order of their class, and indulge in the crapulous appetites and low habits of their new associates. The causes of this vocal peculiarity are owing, Duchatelet thinks, to the excessive use of alcoholic liquors, and to the atmospheric extremes and vicissitudes to which the prostitutes are exposed.

Some *peculiarities* in regard to the *color of the hair, and of the eyes and eyebrows of prostitutes*, are inquired into by the author. Passing over his numerical estimates, we give his conclusions as relates to climatic influences. They are—1. That the varieties of black and auburn hair are more frequently seen in proportion as we pass from the north to the south. 2. That brown hair is less prevalent in the north. 3. That the blonde is more frequently seen as we approach the north from the south. 4. Finally, that red hair follows the same law as the blonde, and is not met with in the southern departments of France. The color of the eyebrows is like that of the hair, and the remarks made on the one will apply to those on the other. This is not the case, however, with the eyes. Of 12,454 women in whom this physiognomical trait was carefully noted, it was observed that the eyes in 4612 were gray; in 3529 brown; in 2878 blue; in 730 red; in 705 black. The proportions of each class in 1000 were, respectively, (omitting decimal parts of a unit,) 870, 283, 231, 38 and 30. Nothing positive could be inferred as to any relation between the color of the eyes and geographical position.

On the question of the *stature* of the prostitutes of Paris, Duchatelet has been enabled to give positive information, derived from documents which had been thrown aside. The original object in collecting them, and the quarter whence he obtained them, are not mentioned. They are exhibited in a series of tables, from which we cull a few items. In 14,126 prostitutes whose stature is recorded in these tables, there was one, from the city, of the height of three feet nine inches; and, on the other extreme of the scale, there was one, from the country, who was six feet and three-quarters of an inch high. If such a woman had met the eye of Frederick William of Prussia, father of Frederick the Great, he would have made, as the phrase is, "an honest woman of her," by having her married to one of his giant grenadiers. The stature of which there were the greatest number,—903, was five feet; after these come 883 who were five feet one inch in height; then 820 and 806 whose stature was, respectively, four feet eleven inches, and five feet and within a few lines of two inches.

*The condition of the sexual organs in prostitutes* is a subject which, apart from any other consideration, is important in its medico-legal bearings. Parent-Duchatelet, with an air of reticence, which is manifest throughout his work, had at first intended not to notice it; but his best friends assured him that, if his investigations were useful, they were, by right, no longer his, but were due to science and justice. He yielded to these opinions, the more readily, perhaps, from the recollection of some facts closely connected with the question proposed. Many years before, as he tells us, two young girls of very decent appearance were accosted in broad day by some young men, who lavished on them the most opprobrious epithets. Persons on the spot sided with the girls, and a complaint was lodged against their traducers, who were summoned before a magistrate. During the discussions to which the affair gave rise the girls insisted on their being virgins, and fearing that they would lose their cause by the weight of arguments on the other side, they expressed their readiness to be examined by a physician appointed by a magistrate, and qualified, on oath, for undertaking the task. The report of the improvised expert, who was an able and conscientious man, was to the effect, that in the case of one of these young women it was impossible for him to give a decided opinion, and as to the other, he thought it not unlikely that she had had connection with men, but he would not venture to speak positively on this point. Duchatelet does not remember what decision was reached on the occasion, but it was afterwards ascertained that these two *soi-disant* virgins had been not only for a long time inscribed on the registers of the police, but had several times contracted the venereal disease. Again, Duchatelet says, that in many instances of alleged rape, he could not gain any more satisfactory information from the details furnished by the young females themselves than from an examination of their persons, and hence he always avoided making any reports on the subject, for fear of compromising the interests of justice. Putting aside popular belief and the slang of old libertines in both the higher and lower walks of society, which imply that the genital organs of prostitutes cannot help exhibiting changes and evidences of a peculiar condition, Duchatelet made careful inquiries of the physicians and surgeons of the Dispensary and of the hospital to which these women, when they fall into the hands of the police, are taken. The result was a unanimous declaration, that prostitutes, in the particulars just stated, exhibited nothing special or peculiar. In the case of young girls who had been engaged in prostitution before reaching puberty, it was found extremely difficult, not to say impossible, to infer their loss of virginity and their mode of life from an examination of their sexual organs. M. Jaquemin, after numerous examinations (4500) of this nature, was led to announce, as an almost certain sign of pregnancy, a violet color of the entire mucous membrane of the vagina. In discussing the present theme, a question comes up respecting the state of the clitoris in prostitutes, among whom it is thought to be larger than in other women. This belief is not, however, borne out by actual observations made on a large scale. The author mentions three cases of extraordinary development of this organ; in one of which menstruation had never taken place, and the mammæ were entirely wanting. This person had become a prostitute to make a livelihood, but she always felt the greatest indifference for the other sex as well as for her own. The other two women, although they had their regular monthly returns and fully developed breasts, expressed themselves in a similar manner respecting the coldness of their feelings for men, and for women likewise. The value of this statement will be felt, when taken in connection with the fact, that, in many instances of erotic passion indulged in by women for individuals of their own sex, as previously adverted to, there was no unusual growth of the clitoris. The appearance of a beard in women is generally believed to be accompanied by less femininity and some approach to the other sex in physical conformation and tastes. Duchatelet states that the three women, already noticed for the large size of their clitoris and want of the warmth of sexual feeling, had no beards; and he did not find in those that had this capillary growth any deficiency in regard to sexual organization or appetite.

Excessive development of the *labia majora* cannot be said to be a peculiarity of prostitutes. The state of the anus is spoken of by the author, as it may be supposed to be modified by indulgence in an unnatural and beastly practice, from which, it is a grievous thing to be told, few of the prostitutes of Paris are exempt. This crime, though not necessarily connected with prostitution, must, as one of its collateral effects, serve to excite disgust at this trade, even on the ground of refined sensuality and pain, viewed morally; and ought to prompt to combined efforts toward its diminution and mitigation, if not its entire cessation.

*Menstruation*, in the degraded class of women whose physiological state we are studying, is, with many individuals, quite regular, in others the reverse; but, notwithstanding the most careful and minute inquiries, Duchatelet was unable to learn the circumstances under which these differences occurred. The excesses of all kinds in which these women indulge, and their exposure to atmospherical vicissitudes, cannot fail to interfere often with the functions peculiar to their sex.

Investigations, both direct and comparative, made on a large scale, authorize Duchatelet to assert that, although prostitutes are far from being as *sterile* as is commonly represented, they are *less productive* than the same number of women who lead a regular life. On one point, akin to this subject, there is unanimity of opinion. It is, the frightful mortality among the recently-born children of prostitutes. Of eight children born in the prison (period not stated) four died in the first two weeks after birth, and four in the course of the first year. Of ten children born in the hospital during a twelvemonth, five died at the very moment of their birth, and the other five before the entire recovery of their mothers. At the Maternité, or great Lying-in Hospital of Paris, in which prostitutes are allowed, but not known as such, to enter and be delivered, their labors are always tedious and difficult, and require the use of the forceps. The children for the most part die.

The *influence of the trade* of prostitution on the general health of those who practice it, is stated by Duchatelet with his habitual care and conscientiousness. The result of his inquiries is, that prostitutes are not more subject than other women to ordinary diseases. Under this head we do not include itch and syphilis, to both of which these unfortunate creatures are peculiarly prone, and from which, especially the latter, they suffer so terribly. Syphilis forms the subject of a separate chapter in the second volume of the present work. Another exception to the general proposition just laid down, is found in uterine hemorrhage, to which, the author believes, they are more subject than other females; this is especially noticeable in very young persons of this class, whereas such hemorrhages are very rare in virtuous girls of equally tender age. Surprise will be felt at a mention of the fact that prostitutes are not more liable than other women in the community to organic diseases of the uterus, including cancer itself. Recto-vaginal fistula, following abscesses in the partition between the two cavities, and which are sometimes caused by neglected chancres, might, putting aside this latter cause, be supposed to afflict prostitutes more than the individuals of their sex who lead a regular life. Strange to say, this infirmity, as painful as it is disgusting, is frequently no obstacle to the woman continuing her trade of prostitution, and even becoming a special favorite in it. The scrofulous constitution is very common in this degraded class, and contributes largely to the crowding of the wards of the Infirmary, and to the aggravation of syphilitic affections. To it we must attribute the incurable ulcers, cutaneous affections, and loss of nose so common in the inmates of that institution. Prostitutes, as we might suppose, *à priori*, are more subject to insanity and suicide than the rest of their sex. As relates to the period spent in the hospital by each individual of one entire class of prostitutes, we learn that it may be rated at ten days in the year.

In all our observations on the diseases of prostitutes, it should be borne in mind that we have not to deal with a class of persons who, once engaged in the trade, continue to ply it during the rest of their life. Prostitution is, on the contrary, a transition state, which its followers try to escape from as soon as they can; and hence, although the ranks are always filled, the persons are continually changing. Three years is, for the majority, the ordinary period of this vile service. But in this time how many are carried off by death or rendered entirely helpless by protracted disease! Where, it will be asked, with a natural feeling of curiosity, do these birds of passage wing their flight and settle down during the rest of their life? Duchatelet devotes to this question a chapter which embodies answers to the inquiries made by him in all quarters. The majority of the prostitutes who do not die while engaged in their trade, attach themselves to old bachelor or widower workmen, especially scavengers and rag-gatherers, and, while passing as legitimate wives, take care of these men, prepare their meals, etc. Many, however, after the death of their companion, being without resources, get themselves inscribed once more, and fall back on their old trade. Some engage in various employments common with the sex,—laundresses, fruit-sellers, dealers in articles of the toilet, etc.; but, at the same time, a number of them act as agents for those who pander to the licentiousness of the other sex, such as keepers of brothels, etc. Some go out to service; some, but in small number, get married; others become kept-mistresses to men who apply in due form at the office of registration to have the names of their favorites erased from the list of inscription. Among these bailers out we find mentioned "seven rich Americans;" whose names of course are not given. Not a few prostitutes addict themselves to robbery, and are associated with the thieves of every kind who swarm in Paris. A small number, repenting of their vicious life, retire into asylums, in which they pass the rest of their days.

Here we must put aside, at least for the present, the volumes of Parent-Duchatelet. We would like, for the benefit of our readers, to continue in his instructive company, and to repeat the prominent passages in his chapters on various phases of prostitution yet unnoticed, as well as on the police and sanitary regulations adopted by the French authorities for the regulation and health of prostitutes; the various establishments in which they live; the hospitals in which they are received for the treatment of disease, and the prisons to which they are committed for crimes. Not only must we pass over these topics, but also leave untouched the separate and more or less full accounts of prostitution in the other great cities of Europe, contributed, as a supplement to the present work, by the able editors, MM. Trebuchet and Poirat Duval. This portion alone amounts to 500 pages. If, hereafter, we should look over the large field of prostitution and its melancholy causes and concomitants, as they are found on English ground, and especially in London, Mr. Acton's volume would be put into active requisition. The condition of things in our own country ought also to engage attention. This can be done more effectually after a knowledge of the details and method of inquiry furnished in the great work of Parent-Duchatelet.

